# Effect of Different Wheat-Based Diets and Corn Meal Addition on Development Parameters of *Ephestia kuehniella* (Zeller, 1879) (Lepidoptera: Pyralidae)

**DOI:** 10.1093/jisesa/ieac029

**Published:** 2022-05-20

**Authors:** L M Parra, A G Garcia, G R Alves, S R Magro, J R P Parra

**Affiliations:** ESALQ/USP, Insect Biology Laboratory, Avenida Pádua Dias, 11, Piracicaba, São Paulo, 13418900, Brazil; ESALQ/USP, Insect Biology Laboratory, Avenida Pádua Dias, 11, Piracicaba, São Paulo, 13418900, Brazil; Research and Development Department, Koppert Biological Systems Brazil, Via Vicente Verdi, 528, Charqueada, São Paulo, 13515000, Brazil; Research and Development Department, Koppert Biological Systems Brazil, Via Vicente Verdi, 528, Charqueada, São Paulo, 13515000, Brazil; ESALQ/USP, Insect Biology Laboratory, Avenida Pádua Dias, 11, Piracicaba, São Paulo, 13418900, Brazil

**Keywords:** biological control, natural enemy, factitious host, optimization

## Abstract

The expansion of Integrated Pest Management (IPM), including biological control, has had several positive consequences for the agricultural environment and participants in the production chain. To enable successful operation and applications of biological control, production of insects used for rearing natural enemies (parasitoids and predators) must be optimized to reduce time and costs and improve production both qualitatively and quantitatively. The present study evaluated the effect of wheat varieties, the main component of artificial diets for *Ephestia kuehniella*, on the reproductive performance and biological parameters of this flour moth, which is used for mass production of *Trichogramma* spp. (Hymenoptera: Trichogrammatidae) and other parasitoids and predators. Four varieties of wheat were compared: BRS 327, BRS Marcante, BRS Parrudo, and KBR, with and without the addition of corn *E. kuehniella* reared on 97% BRS 327 wheat flour + 3% nutritional yeast had the best biological parameters and substitution of corn for about half of the wheat increased the number of eggs per female.

In view of concerns regarding the intense applications of chemicals in agriculture, the use of alternative strategies such as biological control to mitigate pest damage has been growing worldwide. The use of biological control agents is increasing by an estimated 10 to 15% per year worldwide (van Lenteren et al. 2018), but may be increasing even faster in Brazil (Vivian and Querino 2020). Egg parasitoids of the genus *Trichogramma* are the macro-organisms used most often; for instance, in 2021 these wasps were being used on approximately 3 million hectares of sugarcane in Brazil (Parra et al. 2021).

*E. kuehniella* is an important pest in warehouses, where it causes significant damage to stored products. Because it can be reared in the laboratory on a low-cost diet, this moth serves as a factitious host for production of *Trichogramma* spp., reducing production costs and producing longer-lived and more-fertile parasitoids (St-Onge et al. 2015, Moghaddassi et al. 2019, Laumann and Sampaio 2020). The eggs of *E. kuehniella* have also been used for mass production of other natural enemies, such as generalist predators (Calixto et al. 2014).

The diets most often used to rear *E. kuehniella* are composed of 97% wheat flour (*Triticum* spp.) and 3% nutritional yeast (Parra et al. 1989). The food substrate offered to lepidopteran larvae can influence their development and reproduction parameters (Pratissoli et al. 2008), affecting the rearing. Addition of other components to the diet can significantly impact production of the moth. For instance, Kurtuluş et al. (2019) found that diets with wheat flour, wheat bran, glycerin, and nutritional yeast in the proportion of 53.33, 26.67, 15, and 5% respectively shortened the moth’s development time and increased its fertility. The use of different proportions of wheat flour, as described by Tarlack et al. (2014), also affects rearing.

Certain risks are associated with the use of diets composed of common whole-wheat flour because it may contain chemical residues and other contaminants that affect the insects. In addition, the nutritional properties or the particle size of wheat flour may not be attractive to *E. kuehniella* (Locatelli et al. 2008, Tarlack et al. 2014). The use of known varieties of wheat flour may help to solve this problem, but since the nutritional composition, e.g., content of proteins and carbohydrates, differs among varieties, it is necessary to select the variety that is most suitable for the insect.

For *E. kuehniella* specifically, Lima-Filho et al. (2001) found that diets enriched with corn meal resulted in higher viability of the immature stages and higher fecundity of the adults. Although the role of corn meal in *E. kuehniella* diets is unclear, certain sugars in insect diets function as phagostimulants, i.e., attractive components included to stimulate food intake (Davis 1968, Lasa et al. 2009), besides their nutritional aspects. Sugars are a universal metabolic source and are involved in modulating insect responses to secondary compounds (Panzuto et al. 2002, Pentzold et al. 2017).

With this in mind, we compared the nutritional effects of diets composed of whole-wheat flour made from four different varieties of wheat (*Triticum* spp.) with different nutritional compositions, and evaluated their effects on biological and reproductive parameters of *E. kuehniella.* For each variety, we also assessed an additional dietary component, corn meal (*Zea mays* L.). In sum, we investigated whether there is a difference between wheat varieties and whether the presence of corn meal affects the development parameters of *E. kuehniella.* Given the importance of this insect for mass rearing of parasitoids and predators, we conducted this study to select the best diet composition and help to improve the production of *E. kuehniella*. We hoped to identify the most appropriate wheat variety according to the parameters studied here, as well as if the addition of corn meal could improve the current wheat-flour diets.

## Material and Methods

### Selection of Wheat Varieties for Rearing *Ephestia kuehniella* (Zeller, 1879) (Lepidoptera: Pyralidae)

We evaluated the main varieties of nontransgenic wheat available in Brazil and obtained 25 kg of 13 varieties of wheat grains, provided by three companies, *Embrapa Trigo*, *Biotrigo*, and *Agraria Cooperative*. All wheat varieties were cultivated in southern Brazil under similar environmental conditions: *Biotrigo* and *Embrapa Trigo*’s varieties came from Passo Fundo, Rio Grande do Sul State and *Agraria Cooperative’s* varieties came from Guarapuava, Paraná State (Supp. Fig. 1 [online only]). The nutritional properties of the wheat grains were analyzed quantitatively and qualitatively through bromatological analysis at EsalqLab in the Department of Animal Science, Luiz de Queiroz College of Agriculture, University of São Paulo (ESALQ/USP) in Piracicaba, São Paulo. This chemical analysis provided data on the contents of crude protein, fiber, dry matter, and minerals. The four wheat varieties that contained the highest percentages of proteins and carbohydrates were selected (Table 1): BRS 327, BRS Marcante, BRS Parrudo (all provided by *Embrapa Trigo*), and KBR (a combination of the varieties provided by *Biotrigo*: TBIO Toruk, TBIO Sonic, and TBIO Sossego). KBR is prepared by Koppert Biological Systems Brazil, located in Charqueada, São Paulo (Supp. Fig. 1 [online only]). The main components of the corn meal used in some diet mixtures were analyzed at the same laboratory and are given in Table 1.

**Table 1.** Main nutritional components (%) of the diets used in the study

Main components (%)	KBR	BRS 327	BRS Marcante	BRS Parrudo	Corn Meal
Dry matter	89.2	88.2	88.9	88.9	89
Proteins	15.8	17	17.2	18.6	7.8
Fibers	3.8	2.65	4.1	3.5	7.2
Total digestible nutrients	77.2	78.0	76.4	76.6	77.1
Fats	1.5	2.0	1.2	1.5	4.9
Mineral matter	1.8	1.9	2.1	1.9	1.6
Carbohydrates	61.2	63.4	61	60.7	70.8

**Fig. 1.**
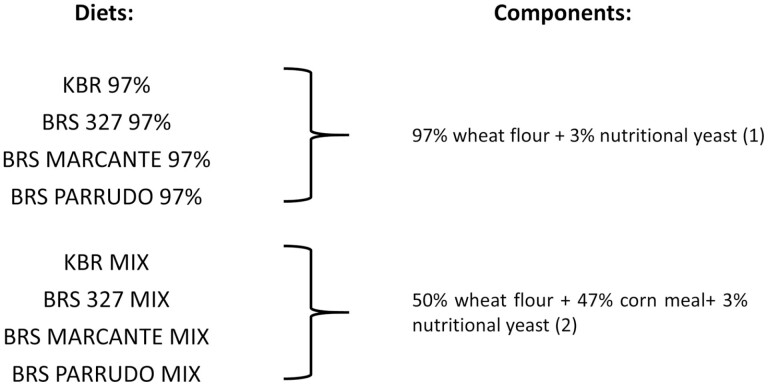
The eight diets used in the experiment; each of four wheat varieties was mixed either with wheat and nutritional yeast or with wheat, corn, and nutritional yeast (‘MIX’).

The diets were prepared at the Research and Development Laboratory of Koppert Biological Systems Brazil, as follows: (1) 97% wheat flour + 3% nutritional yeast (termed ‘97%’) and (2) 50% wheat flour + 47% corn meal + 3% nutritional yeast (termed ‘MIX’), giving eight treatments (Fig. 1). The population of *Ephestia kuehniella* was obtained from the Koppert Biological Systems located in The Netherlands. The company has reared this species for over 10 years according to reliable standardized techniques, at 25 ± 2 °C, 60 ± 10 RH, and 14 hr of photophase.

### Effect of the Diets on *E. kuehniella* Rearing During the First 20 d

The initial survival of *E. kuehniella* larvae was evaluated on the 20th day after the eggs were placed on the diet. The bioassay, with 4 × 2 treatments (4 wheat varieties, with and without addition of corn) (Fig. 1) and three replications each, was performed under laboratory conditions, with controlled temperature (28 ± 2 °C), relative humidity (60 ± 10%), and photophase of 14 h. Six grams of each diet was sieved on Gerbox-type plates, where 180 eggs (egg viability 100%) of *E. kuehniella* were inoculated. The initial survival (from the egg to the 3rd week of larval development) was evaluated and calculated by counting the total number of *E. kuehniella* larvae in each Gerbox, using forceps and a manual counter.

### Effects of Each Treatment on Different Development Parameters of *E. kuehniella* (Egg–Adult Period)

The second bioassay, composed of 4 × 2 treatments and 15 replications, was performed under the same laboratory conditions as the first. Each experimental unit consisted of plastic trays (44 × 30 × 8 cm) containing approximately 1 kg of each diet on a cardboard sheet with grooves (to facilitate pupation), inoculated with 3,600 eggs (approximately 100 mg) of *E. kuehniella*, and covered with voile fabric. Beginning with the emergence of the first adults, the moths from each tray were collected daily for 14 consecutive days. The moths were anesthetized with carbon dioxide (CO_2_) for 2,5 min. The anesthetized moths were carefully removed and placed in oviposition cages (15 cm in diameter). Data for moth weight (individually and total) were calculated to estimate overall survival, development period, and number of eggs per female (see following subsections).

#### Sex Ratio

Four samples of 50 adults each per treatment were collected and sexed based on the terminal portion of the abdomen. After separation by sex, the moths were weighed separately on an analytical balance, obtaining the average weight of each male and female. The sex ratio was calculated as:


$$\begin{array}{l} SR=number\;of\;females/(number\;of\;males\;\;\;+\;\;\;females) \end{array}$$ (1)

#### Overall Survival and Development Period

The oviposition cages were weighed before and after the adults were collected, using a precision balance, in order to estimate the total weight of adults collected per treatment. Based on the average weight of males and females, the sex ratio, and the total weight of adults, we determined the number of moths (*NM*) produced in each tray (Equation 2). The overall survival of the egg-adult period could then be determined, since 3600 eggs of *E. kuehniella* were initially inoculated in each tray.


$$\begin{array}{l} \begin{array}{lll} & NM= & \;\;\;\left(weight\;\;\;of\;\;\;cages\;\;\;with\;\;\;adults\text{-}weight\;\;\;of\;\;\;cages\;\;\;without\;\;\;adults\right)\\ & & \;\times sex\;\;\;ratio\times average\;\;\;weight\;\;\;of\;\;\;females\\ & \;\;\; & \;+\left(weight\;\;\;of\;\;\;cages\;\;\;with\;\;\;adults\text{-}weight\;\;\;of\;\;\;cages\;\;\;without\;\;\;adults\right)\\ & & \;\times (1-sex\;\;\;ratio)\times average\;\;\;weight\;\;\;of\;\;\;males \end{array} \end{array}$$ (2)

The average development period (DP) from egg to adult was calculated by Equation 3. The eggs were inoculated on the same date for all treatments. The total sum obtained was divided by the total weight of adults.


$$\begin{array}{l} \begin{array}{lll} & DP= & (date\;\;\;of\;\;\;collection\;\;\;of\;\;\;adults-date\;\;\;of\;\;\;egg\;\;\;inoculation)\\ & & \;\times (total\;\;\;weight\;\;\;of\;\;\;adults\;\;\;collected\;\;\;on\;\;\;that\;\;\;date) \end{array} \end{array}$$ (3)

#### Number of Eggs Per Female

The eggs from each oviposition cage were collected during four consecutive days. The egg masses were collected with the aid of a brush and each egg mass was stored in a plastic pot and weighed on an analytical balance. The number of eggs per female was calculated by assuming that 1 gram of *E. kuehniella* eggs contains 36,000 eggs (Parra et al. 1989) (Equation 4).


$$\begin{array}{l} Number\;of\;\;\;\;eggs\;per\;female=\frac{\left(total\;weight\;of\;eggs\times 36,000\right)}{number\;\;\;\;of\;adults\times sex\;ratio} \end{array}$$ (4)

### Statistical Analysis

The average development period was tested for homogeneity (Burr and Foster 1972), normality, and independence of the residuals (Shapiro and Wilk 1965). In the case of normality, it was analyzed by ANOVA and compared using Tukey’s test (*P* ≤ 0.05).

To determine how the biological parameters were affected by the different varieties of wheat flour and by the presence of corn meal, we used a generalized linear model (GLM) with a quasi-Poisson distribution for the number of eggs per female, and with a quasi-binomial distribution for the insect’s initial and overall survival, and sex ratio, followed by Tukey’s multiple comparisons test (*P* < 0.05). We assessed the goodness-of-fit using half-normal plots with simulation envelopes for all models (Moral et al. 2017). The number of eggs per female (Equation 4) is continuous, therefore we fitted a Gaussian model, assessing the significance of the effects using F test.

All statistical analyses were carried out using R 3.4.3 (R Development Core Team 2017).

## Results

Overall, the results indicated that addition of corn to the diets did not result in significant increases in the initial survival during the first 20 d, and only affected the overall survival when mixed with BRS Parrudo. On the other hand, addition of corn shortened the development time in all diet varieties. Of all wheat varieties, BRS 327 resulted in the highest number of eggs per female. The results are detailed in the following subsections.

### Effect of the Diets on *E. kuehniella* Rearing During the First 20 d

Over the initial 20 d, the initial survival was not affected by the wheat variety (*F* = 0.55; df = 3,20; *P* = 0.65), by the presence of corn meal (*F* = 3.35; df = 1,19; *P* = 0.09), or by the interaction between these two factors (*F* = 0.14; df = 3,16; *P* = 0.94), ranging between 43.0% and 63.9%.

### Effects of Each Treatment on Different Development Parameters of *E. kuehniella* (Egg–Adult Period)

#### Sex Ratio

The sex ratio was not affected by the presence of corn meal (*F* = 0.7865; df = 3,28; *P* = 0.58), the wheat variety (*F* = 3.867; df = 1,27; *P* = 0.06), or the interaction between these factors (*F* = 2.55; df = 3,24; *P* = 0.08), remaining close to 1:1.

#### Overall Survival and Development Period

Overall survival was significantly affected by the variety (*F* = 6.0801; df = 3.32; *P* < 0.01) and by the presence of corn meal only for BRS Parrudo (*F* = 5.4239; df = 1,31; *P* < 0.01), but not by the interaction between variety and diet (*F* = 1.3708; df = 3,28; *P* = 0.27). Overall, the diets based on varieties BRS Parrudo and BRS 327 resulted in higher survival (Table 2).

**Table 2.** Overall survival (%) during the development of *Ephestia kuehniella* reared on different diets

Wheat variety	Overall survival (%)	
	97%^*a*^	MIX^*b*^
BRS 327	82.6 ± 6.9Aa	80.0 ± 5.8Aa
KBR	50.4 ± 3.7Abc	62.2 ± 13.2Aa
BRS Parrudo	73.3 ± 3.7Aac	89.9 ± 4.0Ba
BRS Marcante	37.1 ± 18.2Ab	73.5 ± 9.4Aa

Means followed by the same lower case letters in columns and upper case in rows do not differ at the 5% level.

^*a*^‘97%’ composed of 97% wheat flour + 3% nutritional yeast.

^*b*^‘MIX’ composed of 50% wheat flour + 47% corn meal + 3% nutritional yeast.

The life cycle was affected by both the diet (*F* = 439.62; df = 1,28; *P* < 0.001) and the variety (*F* = 15.879; df = 3,28; *P* < 0.001). The cycle was longer for the 97% than for the MIX diet (Table 3). Regarding the variety, the longest periods were observed for moths on the diets based on BRS Parrudo and BRS Marcante wheat for the 97% diet, and BRS 327 and KBR wheat for the mix diet.

**Table 3.** Egg-adult development period (days) of *Ephestia kuehniella* reared on different diets

Wheat variety	Egg-adult development period (days)	
	97%^*a*^	MIX^*b*^
BRS 327	61.9 ± 0.149Aa	59.1 ± 0.103Ba
KBR	61.7 ± 0.244Aa	58.2 ± 0.580Ba
BRS Parrudo	67.3 ± 0.408Ab	57.4 ± 0.239Bb
BRS Marcante	66.9 ± 1.060Ab	56.9 ± 0.600Bb

Means followed by the same lower case letters in columns and upper case in rows do not differ at the 5% level.

^*a*^‘97%’ composed of 97% wheat flour + 3% nutritional yeast.

^*b*^‘MIX’ composed of 50% wheat flour + 47% corn meal + 3% nutritional yeast.

#### Number of Eggs Per Female

Only differences between the wheat varieties were observed (*F* = 4.0095; df = 3,32; *P* < 0.05), with females laying the most eggs on varieties BRS 327, KBR, and BRS Marcante for the 97% diet, and BRS 327 and BRS Marcante for the mix diet (Table 4).

**Table 4.** Number of eggs laid by females of *Ephestia kuehniella* reared on different diets

Wheat variety	Number of eggs per female	
	97%^*a*^	MIX^*b*^
BRS 327	255 ± 9.28Aa	270 ± 5.20Aa
KBR	216 ± 19.1Aab	244 ± 16.6Ab
BRS Parrudo	177 ± 19.7Ab	215 ± 4.65Ab
BRS Marcante	185 ± 70.6Aab	249 ± 7.08Aab

Means followed by the same lower case letters in columns and upper case in rows do not differ at the 5% level.

^*a*^‘97%’ composed of 97% wheat + 3% nutritional yeast.

^*b*^‘MIX’ composed of 50% wheat + 47% corn + 3% nutritional yeast.

## Discussion

The addition of corn did not influence the initial survival during the first 20 d, and only increased overall survival for the variety BRS Parrudo in the mix diet (Table 2), differently from other studies that found that corn stimulated food intake due to the presence of carbohydrates (sugars) (Reinecke 1985; Lima-Filho et al. 2001). Sugars such as sucrose, fructose, and glucosides have been reported as phagostimulants for phytophagous insects, because these ingredients can stimulate the insect’s feeding, survival, and development and increase its ability to establish a colony (Davis 1968). Regarding BRS Parrudo, in the mix diet, the increase in overall survival could be due to a limited amount of carbohydrates, since this variety is lowest in carbohydrates (Table 1). On the other hand, the presence of corn resulted in faster development, most notably for BRS Parrudo and BRS Marcante on the mix diet (Table 3). The reduced development time may be related to phagostimulation, since stimulating the consumption of a diet benefits insect growth (Davis 1968). Phagostimulant effects may occur for different reasons; for instance, the granulometry and texture influence palatability. Locatelli et al. (2008) observed larger numbers of *E. kuehniella* adults in wheat flours with a grain size of 250 µm than in flours with a grain size of 419 µm. Kurtuluş et al. (2019) found that addition of glycerin to corn-based diets could affect the texture, reducing pupation and emergence of adults of *E. kuehniella.*

The benefits of corn as a phagostimulant may be also related to a higher intake of proteins. Seyedi et al. (2017) found that protein levels in the midguts of 5^th^-instar larvae were higher in larvae reared on wheat-based diets with added corn than in larvae reared on pure wheat-based diets, although the protein compositions of the two diets were similar. This demonstrates the importance of corn as a phagostimulant in diets and shows that the nutritional composition is not the only important factor to consider when insects are reared in artificial conditions (Kurtuluş et al. 2019).

The addition of corn resulted in shorter mean development period for all wheat varieties (Table 3). Kurtuluş et al. (2019) showed that addition of several components to diets, such as nutritional yeast and wheat bran, can shorten the development time of *E. kuehniella* and increase its fertility because of the correct balance of nutrients and minerals. Addition of wheat bran, glycerin, and yeast to *E. kuehniella* diets gave similar results, significantly shortening the development time compared with diets based only on corn meal and yeast (Pehlivan 2021).

Lima-Filho et al. (2001) observed that *E. kuehniella* reared on diets with corn meal tended to have shorter development periods, with a minimum of 38 d. Similarly, Ayvaz and Karabörklü (2008) showed that *E. kuehniella* larvae developed more rapidly to the adult stage when reared on a corn flour diet. According to them, the delayed development is related to the lower protein and carbohydrate contents of a diet based on wheat bran when corn is absent.

Vasconcelos (2017) found no significant differences in the number of eggs per female between treatments with different varieties of wheat. Females laid 300 eggs on average, more than observed in the present study. This difference probably occurred due to the large-scale rearing method used here, such as that adopted by the company (Koppert Biological Systems Brazil) where this study was conducted. An increase of lipids in the diet can also raise *E. kuehniella* reproduction rates, resulting in larger and more viable eggs (Moghaddassi et al. 2019), and addition of yellow corn can also increase fecundity (Magrini et al. 1995).

Adult parameters, such as body size, development, and female fecundity are influenced by the diet (Gianoli et al. 2007; Altermatt 2010), particularly in insects such as *E. kuehniella* that feed only in the larval stage, when they accumulate the nutrients necessary for the adult stage (Bauerfeind and Fischer 2005, Maciá 2009, Kurtuluş et al. 2019). The quality of the host is one of the most important factors in rearing parasitoids (Charnov et al. 1981, Werren 1984, Wajnberg 2009). A balanced diet that meets nutritional requirements can positively affect its development, especially in *E. kuehniella.* (Farahani et al. 2016).

In conclusion, the statistical analysis indicated that when no corn is added (97%), BRS 327 diet supported greater overall survival, along with KBR a shorter egg to adult development time, and oviposited statistically the same high number of eggs as KBR and BRS Marcante. When corn was added (MIX), the egg to adult development time for BRS 327 decreased by 3 d. Thus, BRS 327 is the preferred wheat variety for the 97% diet and also is suitable for use in the MIX diet for rearing *E. kuehniella.*

## Supplementary Material

ieac029_suppl_Supplementary_Material
