# Supplementary material for: Effect of Different Wheat-Based Diets and Corn Meal Addition on Development Parameters of Ephestia kuehniella (Zeller, 1879) (Lepidoptera: Pyralidae)
Source: J Insect Sci. 2022 May 20;22(3):10. doi: 10.1093/jisesa/ieac029 (PMC9123304; doi:10.1093/jisesa/ieac029)
Supplement: ieac029_suppl_Supplementary_Material [file ieac029_suppl_supplementary_material.docx]

**
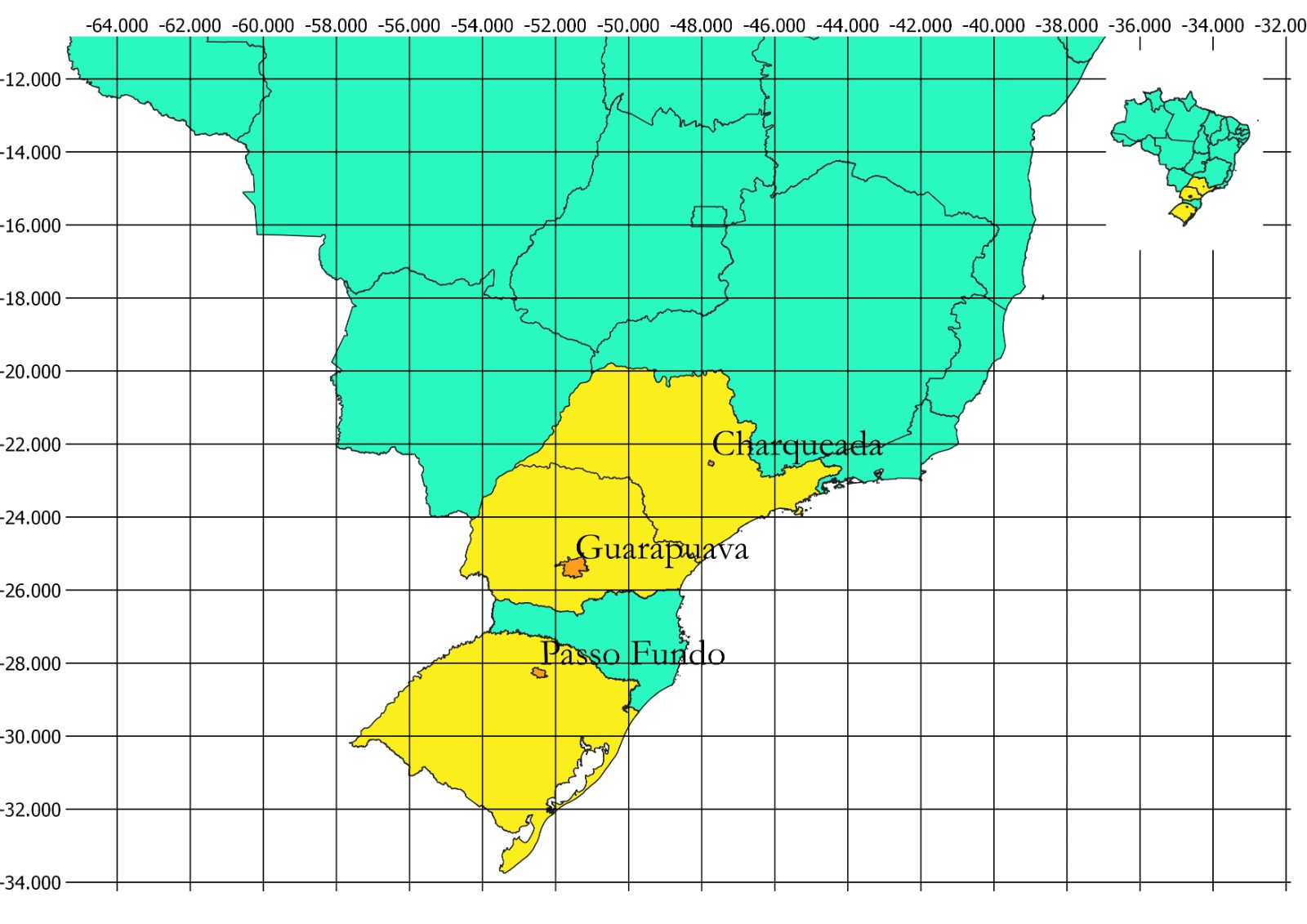
Supp. Figure 1.** Locations of the companies that contributed to this work. From top to bottom: Koppert (Charqueada, São Paulo state); the Agraria Cooperative (Guarapuava, Paraná state); Company Biotrigo and Embrapa Trigo (Passo Fundo, Rio Grande do Sul state)
